# Ticks and Tick-Borne Infections: Complex Ecology, Agents, and Host Interactions

**DOI:** 10.3390/vetsci5020060

**Published:** 2018-06-20

**Authors:** Stephen K. Wikel

**Affiliations:** Department of Medical Sciences, Frank H. Netter, M.D., School of Medicine, Quinnipiac University, Hamden, CT 06518, USA; stephen.wikel@quinnipiac.edu; Tel.: +1-203-626-9231

**Keywords:** ticks, tick-borne diseases, emerging and resurging pathogens, zoonoses, vector ecology, climate change, tick saliva, tick-host-pathogen interactions, host immune defenses, immunomodulation

## Abstract

Ticks transmit the most diverse array of infectious agents of any arthropod vector. Both ticks and the microbes they transmit are recognized as significant threats to human and veterinary public health. This article examines the potential impacts of climate change on the distribution of ticks and the infections they transmit; the emergence of novel tick-borne pathogens, increasing geographic range and incidence of tick-borne infections; and advances in the characterization of tick saliva mediated modulation of host defenses and the implications of those interactions for transmission, establishment, and control of tick infestation and tick-borne infectious agents.

## 1. Introduction

Zoonotic diseases are a significant negative impact upon global public health that is increasing. Zoonotic diseases are defined as infections shared in nature between humans and other vertebrate animal species [1], characterized by transmission between species of organisms infecting humans that are enzootic in other animal species [2]. Mutually transmissible infections between humans and other animal species are thought to have arisen with the development of agriculture and the resulting contact of more densely populated human communities with pathogens of domesticated and wild animal species [3]. Greater than 60% of human infectious diseases emerging between 1940 and 2004 were zoonotic, resulting in significant global morbidity, mortality, and economic costs [4]. Of those emerging zoonoses, 71.8% are from wildlife and 22.8% are arthropod vector-borne infections [4]. Significantly, the frequency of emerging vector-borne zoonoses has increased during the last ten years [4].

Among arthropod vectors of disease, ticks transmit the most diverse array of infectious agents and ticks are the most important arthropod vectors, globally, of pathogens to humans and domestic animals [5,6,7]. Tick-borne infections of humans are zoonoses of wildlife origins, similar to tick transmitted diseases of companion and domestic animal species [8]. Dynamic interactions among biotic and abiotic elements influence tick-borne disease epidemiology and ecology. Seminal studies by Pavlovsky [9] advanced the concept that zoonotic pathogens and their vectors occur in distinct habitats, resulting in the concept of the natural nidality, or landscape epidemiology, of transmissible diseases. Changes in tick distribution and abundance, as well as emergence, resurgence, and geographic spread of tick-borne infections, are influenced by tick and tick-borne pathogen demography, micro and macro climate changes, human behavior, travel, land use and habitat modification (agricultural, residential, recreational), economics, politics, population growth and movement, and intrinsic changes in ticks and tick-borne pathogens [7,8,10]. The importance and awareness of the impacts of tick-borne diseases are steadily increasing [11].

Defining tick-host-pathogen interactions, at the cellular and molecular levels, are additional areas of study essential for characterizing pathogen transmission, establishment, pathogenesis, and for identifying novel checkpoints for control of both vectors and pathogens. A research focus where advances are being achieved at an increasing pace is the characterization of tick saliva and its ability to modulate multiple host defenses differentially during blood feeding, resulting in successful acquisition of a blood meal and creation of environments favorable for pathogen transmission and establishment in the host [12,13,14,15,16]. This area of study is an excellent example of the effective use of genetics, genomics, functional genomics, proteomics, and the application of a broad array of molecular biology tools to rapidly advance the understanding of complex pathways operating at the tick-host-pathogen interface.

Our understanding of ticks and tick-borne diseases is changing on many levels due to the application of significant technological advances and new paradigms. An avenue for advancing efforts to achieve effective control of ticks and tick-borne diseases is by implementing coordinated planning, performance, and evaluation of diverse research initiatives and integrated control measures in a One Health approach to these vectors and infections of human and veterinary public health importance [11]. The One Health concept is the contemporary version of the coordinated and collaborative human and veterinary medicine approach to zoonoses articulated by Schwabe [1].

This review examines selected contemporary topics of tick and tick-borne disease epidemiology, tick biology, and tick-host-pathogen interactions that are of increasing importance for defining underlying factors, relationships, and mechanisms that are essential for the success of these important vectors and the infections they transmit. Long term objectives of these evolving areas of investigation are to add to the accumulating body of knowledge that will result in the development of effective disease prediction, prevention, and control interventions.

## 2. Changing Ticks and Tick-Borne Diseases

Ticks, tick transmitted infections, hosts, and a multitude of factors that influence them are constantly undergoing change. This constant state of flux for multiple factors impacts: Vector and disease surveillance, reporting, public awareness, and interventions; fluctuations in tick population densities and range at local and national levels; introduction of tick-borne diseases into new areas, resurgence, and emergence within established geographic areas; and the development of surveillance and diagnostic tools to educate healthcare providers and raise awareness of the public both to old and new public health threats. Understanding tick-borne zoonoses requires comprehensive examination and increasing knowledge of the complex associations among tick populations, habitat landscapes, climate, human behavior, human demographics, economics, and intrinsic pathogen factors [10,17,18,19].

Arthropod disease vectors and their transmission of disease causing agents are significantly influenced by weather and climate [20]. Warming global temperatures will influence geographic range and population expansion of ticks, which, in turn, influences distribution patterns and incidences of tick-borne infections [10]. Ticks and tick-borne diseases are predicted to move poleward, with accompanying contractions in subtropical or tropical equatorial ranges [21]. In the northern hemisphere, warmer falls, winters, and springs can, potentially, increase the geographic range of ticks further to the north, as well as to higher altitudes [10]. Likewise, elevated temperatures in other regions could create environments not favorable for the development or survival of some tick species. An environmental temperature rise of 2 °C is predicated to make habitats less favorable for several tick species in South Africa [22]. Since ixodid ticks are particularly sensitive to humidity levels [23,24], the combination of increased temperature and drier seasons could negatively impact tick populations [25]. Ticks are particularly sensitive to variations in rainfall. Due to the complex interactions of ticks, pathogens, reservoir hosts, and weather, any climate changes are likely to influence tick-borne zoonoses more than vector-borne infections that are directly transmitted between humans [26]. The impact of climate change on ticks and tick-borne diseases will be determined over time; however, since differing views exist on the development of needed predictive models, this will be a challenging task [18].

## 3. Ticks on the Move

Changes in geographic distributions of tick populations have, are, and will continue to occur. Modifications of any of the known interacting factors that influence tick populations have the potential to affect the distribution and abundance of these important disease vectors. Simultaneous changes are taking place among those complex associations of factors at micro and macro levels, which require heightened surveillance of ticks and tick-borne diseases that are established, resurging, or emerging.

The northward range expansion of *Ixodes ricinus* in Sweden and Russia, along with an increased abundance of this species at the eastern limits of its range in the Tula region of Russia, are attributed to climate changes, with resultant milder winters [27,28]. *Ixodes ricinus* is the most frequently reported tick in the United Kingdom with an expanding range in southern England [29]. Both *Ixodes ricinus* and *Borrelia burgdorferi* are expanding their range into the northern latitudes of Europe [30]. These findings support the hypothesis that climate change will increase the habitat of *Ixodes ricinus* in northern Europe and Eurasia [31]. *Ixodes persulcatus* and tick-borne encephalitis expansion into the subarctic regions of European Russia are also linked to climate change [32].

The geographic range extension, population increases, and an expanding array of transmitted infectious agents of *Ixodes scapularis* are extensively studied in both the United States and Canada [33,34]. *Ixodes scapularis* geographic range has expanded significantly in the Eastern and Midwestern United States during the past 20 years [35]. Concomitant with this expanded range is an increase in the incidence of reported cases of Lyme disease and other *Ixodes scapularis* vectored pathogens [36]. Between 1996 and 2016, the number of counties in which *Ixodes scapularis* is established doubled to 44.7% of all United States counties [35]. The potential geographic range of this tick exceeds the currently described distribution within the United States [37]. *Ixodes scapularis* range grew into eastern and central Canada by approximately 2004 and it was accompanied by the emergence of Lyme disease [34]. *Ixodes scapularis* is considered to be reclaiming its historical geographic range in response to changes that include habitat and climate changes, as well as the availability of hosts for all life cycle stages, particularly white tailed deer [33]. *Ixodes scapularis* is a competent vector and *Peromyscus leucopus* is a reservoir for an increasing number of human pathogens [33,36], highlighting the public health importance of this tick.

*Dermacentor reticulatus* is a species whose biology is well suited to survive and thrive in diverse, challenging, and changing weather conditions, climates, and habitats in Europe and Eurasia [38]. This tick recently expanded its range into many regions of Europe, raising the potential for the spread of pathogens into these regions [38]. *Dermacentor reticulatus* range extended, during recent decades, into Germany, the Netherlands, Poland, and other European regions that were previously considered to be suitable habitats for this tick vector [39]. Climate and habitat modifications are important factors in geographic range changes for disease vectors and pathogens [40]. Increasing numbers of wildlife hosts and reforestation are factors in the range expansion of this tick [41]. Although *Dermacentor reticulatus* has many possible host species, movement of dogs as companion animals and strays is an important factor in the movement and establishment of this tick in new regions [38]. *Dermacentor reticulatus* is a competent vector for pathogens of human and veterinary public health importance, including: Omsk hemorrhagic fever virus, tick-borne encephalitis virus, *Rickettsia raoultii*, *Rickettsia slovaca*, *Anaplasma marginale*, *Babesia canis*, *Babesia caballi*, and *Theileria equi* [38].

*Amblyomma americanum* is a North American tick of increasing importance due to its significantly expanding geographic distribution, increased population density, and roles as a vector of established and emerging infectious agents [42]. Contributing to the medical importance of *Amblyomma americanum* is the ability of larvae, nymphs, and adults to readily seek and blood feed on humans [42,43]. The distribution of this important pest tick was, historically, from the southeastern United States to west central Texas and north to Iowa; however, the geographic range now extends into the Mid-Atlantic States and New England, and as far north as Maine [44]. The geographic range and population resurgence of white tailed deer are significant, since they are hosts for larvae, nymphs, and adults of this important tick species [45]. This tick species is increasingly recognized as an important vector of infectious agents. White tailed deer are reservoirs for *Amblyomma americanum* transmitted *Ehrlichia chaffeensis* and *Ehrlichia ewingii* [36]. *Amblyomma americanum* is a competent vector for *Francisella tularensis* [36] and is implicated as the vector for the recently discovered Heartland [46,47] and Bourbon viruses [48]. Transmission of the enigmatic southern tick associated rash illness, STARI, or also known as Masters disease, is linked to *Amblyomma americanum* [49].

The human health impact of ticks is evolving along the multiple fronts of infectious agents, tick paralysis, damage due to feeding, and the emergence of allergies to tick saliva molecules. First reported observations linking ticks to red meat allergy occurred in 2007 [50]. Subsequently, delayed onset anaphylaxis following consumption of red meats in patients with a history of tick bites were reported from multiple geographic regions, and the reactions were proven to be due to IgE reactive with galactose-alpha-1,3-galactose, alpha-gal, a blood group oligosaccharide in non-primate mammals [51,52]. Ixodid tick bites linked to the induction of alpha-gal delayed onset anaphylaxis are those of *Amblyomma americanum* [52] and *Ixodes ricinus* [53]. Glycosylation of tick saliva proteins is important for their biological activity. Therefore, eukaryotic expression systems are used to produce recombinant tick saliva proteins [54]. Tick saliva composition and differential expression of saliva molecules during the course of blood feeding are highly complex and modulate many aspects of host immune defenses [12,13,14,15,16].

The range of *Amblyomma maculatum*, the Gulf Coast tick, within the United States has increased significantly since the first half of the twentieth century [55,56]. This emerging vector species of medical and veterinary importance was, initially, limited to the Gulf of Mexico coast from Texas to the Atlantic Ocean coast to South Carolina and as far as 150 miles inland along this zone. Currently, *Amblyomma maculatum* can be found as far north along the Atlantic coast as Delaware, with a range of greater than 250 miles inland, and populations also established in several Midwestern to southwestern states [55,56]. *Amblyomma maculatum* is the principal vector of the spotted fever agent, *Rickettsia parkeri*, and the canine apicomplexian, *Hepatozoon americanum* [57]. Recently, an East Asian tick, *Hemaphysalis longicornis*, was detected as an invasive species in New Jersey and within the past few weeks (June 2018) was also detected in Virginia (http:outbreaknewstoday.com/longhorned-tick-found-cattle-virginia-farm-43455/; https://entomologytoday.org/2018/04/24/invasive-tick-persists-new-jersey/). The means by which this tick arrived in North America remains unclear; however, persistence is a likely possibility after successfully overwintering in New Jersey and spreading to a distant site.

Geographic ranges and abundance of ticks are changing in response to multiple factors that may be operating together to bring about the observed variations [36,56]. Both tick population densities and distributions are impacted by a dynamic environment of biotic and abiotic factors that include changes in human demographics and behaviors. In addition to their direct effects on ticks, biotic and abiotic factors influence the vast number of tick associated microorganisms and tick-borne diseases, as well as the additional public health threats of tick paralysis and other tick toxicoses [58,59].

The complex, ever changing nature and distributions of tick vectors and tick-borne diseases requires continuous surveillance that encompasses the analysis of tick vectors, pathogens, reservoirs, and epidemiology of infections [36]. Public health responses to vector-borne diseases are enhanced by the integration of multidisciplinary teams engaged in the surveillance of vectors and vector-borne diseases, diagnosis, control response strategies, education, research, training of research and operational professionals, and community outreach [60]. Since ticks and tick-borne pathogens do not recognize international boundaries, any network addressing ticks and tick-borne infections must be part of a robust international disease monitoring network that provides timely evidence-based communications to public health officials, healthcare providers, other decision makers, stakeholders, and the public [61,62]. An integrated surveillance and response system that addresses all medically important vectors is most effective and results in significant cost savings over specific vector and related diseases approaches [63].

## 4. Newly Recognized Pathogens Join the Mix

Vector-borne diseases account for a significant proportion of emerging infectious diseases globally [4]. Ticks transmit an increasingly diverse array of infectious agents around the world [64,65]. In the United States, approximately 95% of reported vector-borne diseases are tick transmitted [66]. Conventional methods for identifying causative agents of a tick-borne disease are to associate a specific illness with a history of a tick bite followed by linking that illness to a specific microorganism. A classic example of that process of pathogen discovery is Lyme borreliosis [67]. Early epidemiologic studies provided evidence that a tick, identified as *Ixodes scapularis*, transmitted the agent responsible for the illness [68]. Subsequently, the tick was shown to transmit the causative *Borrelia burgdorferi* spirochetes [69]. Awareness of the increasing complexity of tick associated microorganisms is due to the application of genomics, functional genomics, next generation sequencing, and proteomics to the analyses of tick microbiomes [65,70]. Molecular techniques are now used to reverse pathogen discovery by applying these powerful tools for the identification of previously unrecognized microorganisms in ticks prior to the association of those microbes with a disease [71]. Molecular techniques were the basis for reversed discovery of the following microbes, later established to be human pathogens: *Borrelia miyamotoi* [72], *Neoehrlichia mikurensis* [73], *Rickettsia helvetica* [74], and other *Rickettsia* species [71]. A challenge posed by these powerful and sensitive approaches is to determine which microbes are tick symbionts, disease causing agents, or have the potential to become emerging pathogens of human and veterinary importance.

Here, I examine emerging and resurging tick-borne pathogens, highlighting the diversity of infectious agents and implications of changing tick geographic distributions for changing disease endemic regions. These changes have important implications for vector and disease surveillance, interventions, and education of health care providers and the public.

Among tick vectors of public health importance, *Ixodes scapularis*, and the infectious agents it transmits, are the foci of considerable attention. For an ixodid species that was not considered to be a significant vector of human disease prior to 1970, the diversity of *Ixodes scapularis* transmitted human pathogens stands at seven as of 2017 [33]. Lyme borreliosis is the most commonly encountered *Ixodes scapularis* transmitted disease in the eastern and midwestern regions of the United States [67] and *Ixodes ricinus* transmitted infection in Europe [75]. The estimated number of Lyme borreliosis cases in the United States is in excess of 300,000 [76]. An excellent review of Lyme borreliosis is that of Steere et al. [67]. Likewise, Lyme borreliosis in companion animals is addressed by Krupka and Straubinger [77].

In addition to well established diseases, multiple emerging tick transmitted pathogens were described during the past two decades [33,34,35,36,37,38,39,40,41,42,43,44,45,46,47,48,49,50,51,52,53,54,55,56,57,58,59,60,61,62,63,64,65,66,67,68,69,70,71,72,73,74,75,76,77,78]. The global scope of emerging tick-borne disease causing agents is impressive, including *Babesia* [79,80,81], rickettsioses [71], ehrlichiosis and anaplasmosis [82,83,84], *Borrelia* [78,85], and viruses [86,87].

Piroplasmorida, of the phylum, Apicomplexa, contain *Babesia* and *Theileria*, highly important tick-borne parasites of domestic and wild animal species [88]. Babesiosis is considered the, economically, most important vector-borne disease of cattle [89]. Although wildlife species’ infections with *Babesia* are common, most cases are subclinical [90]. Babesiosis is an increasing global animal health problem. which is partially due to the changing geographic ranges of tick vectors that are occurring, in part, as a result of climate change [91]. There are more than one hundred *Babesia* species that infect a wide range of vertebrate hosts, including fifteen recently described species [89]. New *Babesia* species are reported from regions not previously associated with babesiosis [92]. The assertion has been made that if they are hosts for competent vector ticks, essentially, all vertebrates may be susceptible to *Babesia* infection [89]. The molecular phylogeny, classical and molecular taxonomy, and population genetics of *Babesia* were recently reviewed along with an examination of babesias infecting a variety of unexpected or non-traditional host species from around the world [89].

Babesiosis is a global emerging zoonosis of increasing importance [92]. During the past fifty years, the incidence of human babesiosis increased exponentially [93], a situation likely to continue with the discovery of additional *Babesia* species infecting humans [89]. *Babesia* species that cause disease in humans are *Babesia microti*, *Babesia microti*-like organisms, *Babesia duncani*, *Babesia duncani*-like organisms, *Babesia divergens*, *Babesia divergens*-like organisms, and *Babesia venatorum* [80].

*Babesia divergens* is a bovine parasite that is associated with the infection of splenectomized humans [94]. The first reported human babesiosis case was a fatal *Babesia divergens* infection of a splenectomized individual [93]. *Babesia divergens* infections are most commonly associated with splenectomized individuals, other immunosuppressive conditions, and patients of advanced age [79,80,93]. *Babesia divergens* human infections are occasionally reported in North America and Europe [79,89,93], and, sporadically, in Africa and Asia [95]. Human babesiosis cases were also reported in Australia and South America [80].

The first human babesiosis case occurring in an immunocompetent individual was a *Babesia microti* infection reported in 1969 following the bite of *Ixodes scapularis* [96]. During the years since that initial report, both the incidence and geographic range of *Babesia microti* infections has increased significantly across the northeastern and upper Midwestern United States, with *Ixodes scapularis* infection rates of approximately 20% in well-established endemic areas [80,97]. The expanding range of *Ixodes scapularis*, combined with the current presence of Lyme borreliosis, increases concerns about the emergence of babesiosis in Canada [98]. *Babesia microti* and *Babesia microti*-like organism infections are also reported from Europe, Taiwan, and Japan [80,89,93]. *Babesia microti* is a species complex comprised of three distinct clades [99]. Phylogenetic analysis suggests that *Babesia microti* should be a distinct genus within the Apicomplexa [80].

The family, Anaplasmataceae, within the order, Rickettsiales, contains the tick transmitted genera, *Anaplasma* and *Ehrlichia*, that are important pathogens of companion, domestic, and wildlife species, as well as zoonoses of public health importance [83,100]. The evolving complexity of relationships among the Anaplasmataceae is reflected in the cluster, “*Candidatus Neoehrlichia* species” [83], and reclassification of the genus, *Anaplasma* [101].

Human granulocytic anaplasmosis was originally described in the upper Midwestern United States and assigned the designation of human granulocytic ehrlichiosis, caused by *Ehrlichia phagocytophilum* [84,102,103]. Reclassification of the granulocytic group, *Ehrlichia*, resulted in the change to *Anaplasma phagocytophilum* [104]. In addition to infecting humans, *Anaplasma phagocytophilum* causes tick-borne fever in ruminants, equine anaplasmosis, and febrile illness in cats and canines [83]. Competent vectors of *Anaplasma phagocytophilum* are ixodid ticks of the *Ixodes ricinus* complex; *Ixodes ricinus* in Europe, *Ixodes persulcatus* in Asia, and *Ixodes scapularis*, *Ixodes pacificus*, and *Ixodes spinipalpis* in North America [104]. The number of human anaplasmosis cases has consistently increased since the infection became nationally reportable [84], with a twelve-fold increase between 2001 and 2011 [103]. Human granulocytic anaplasmosis coinfections with other *Ixodes scapularis* transmitted pathogens are an important consideration, with approximately 10% of infected individuals having antibodies that are reactive with *Borrelia burgdorferi* or *Babesia microti* [105]. An expanding range and numbers of vector tick species makes it likely that the incidence of human granulocytic anaplasmosis will continue to increase in the northern hemisphere.

*Ehrlichia* are tick-borne Gram-negative members of the family, Anaplasmataceae, that are of human and veterinary public health importance [83,84]. *Ehrlichia* species with recognized zoonotic potential include: *Ehrlichia chaffeensis*, *Ehrlichia ewingii*, *Ehrlichia canis*, *Ehrlichia muris*, *Ehrlichia ruminantium*, and *Ehrlichia mineirensis* [84]. In addition, new genetic variants of *Ehrlichia*, “*Candidatus Neoehrlichia* species,” were described in Europe, eastern Russia, China, and Japan [83]. Heartwater, caused by *Ehrlichia ruminantium*, is a notifiable disease of importance in domestic and wild ruminants in Africa and regions of the Caribbean [106].

*Ehrlichia chaffeensis*, a causative agent of human monocytic ehrlichiosis, was first described as a human pathogen in 1986 in a seriously ill man with a history of tick bites from the central United States [82]. The enzootic cycle of *Ehrlichia chaffeensis* infection in the United States involves the white tailed deer reservoir and the tick vector, *Amblyomma americanum*, for which all life cycle stages aggressively feed on humans [82]. *Ehrlichia chaffeensis* is the most commonly reported human ehrlichiosis followed by *Ehrlichia ewingii* infection [84,107]. Importantly, *Ehrlichia ewingii* infection occurs in white tailed deer, canines, and ticks throughout the geographic range of *Amblyomma americanum* [108]. The significantly expanding range of *Amblyomma americanum* [55,56], and its role as a vector of *Ehrlichia chaffeensis* and *Ehrlichia ewingii*, suggests that these zoonotic pathogens will also be occurring in new areas, a situation of importance to healthcare providers, public health officials, and veterinarians.

Tick-borne rickettsial diseases have increased dramatically during the past thirty years for established, re-emerging, and emerging pathogens, some of which were previously identified, but not associated, with human disease [71,78]. Tick transmitted rickettsioses are members of the spotted fever group, rickettsiae [109]. Molecular, genomic, and functional genomic tools are the drivers that resulted in the identification of novel species and establish, more clearly, the relationships among rickettsiae, tick vectors, and hosts [110]. An example of the application of these tools is molecular typing, used to identify new *Rickettsia conorii* subspecies *conorii* and *israelensis* [111]. An in depth, comprehensive review of rickettsioses based upon their geographic occurrence was provided by Parola et al. [71] and updated by Kernif et al. [78].

*Borrelia burgdorferi* infection is the topic of numerous studies, as well as the subject of excellent reviews [67]. Lyme borreliosis is increasing in Europe [30,112]. In addition to *Borrelia burgdorferi* sensu stricto, *Borrelia afzelii*, *Borellia garinii*, *Borrelia bavariensis*, and *Borrelia spielmanii* cause human disease [113]. *Borrelia mayonii* was discovered to be an additional causative agent of Lyme borreliosis in the upper Midwestern United States in 2016 [114]. *Ixodes scapularis* is a competent vector for *Borrelia mayonii* [115]. The geographic extent and complexity of Lyme borreliosis spirochetes is increasing across the northern hemisphere range of the *Ixodes ricinus* complex due to the application of new phylogenetic analyses [85]. The clinical importance of the newly identified, tick-borne *Borrelia* species, coupled with an expanding range of vectors, will further evolve and be defined in coming years.

New uncultured *Borrelia* species are reported from Tanzania, Morocco, Ethiopia, and Algeria [78]. *Borrelia miyamotoi* was first described in *Ixodes persulcatus* ticks in Japan; however, it was not associated at that time with human disease [116]. *Borrelia miyamotoi* was subsequently identified as the causative agent of an *Ixodes* tick transmitted relapsing fever [117]. *Borrelia miyamotoi* occurs across the North American, European, and Asian regions where Lyme borreliosis is found [78]. Characterization of the microbiomes of diverse tick species will likely result in the identification of novel tick-borne bacteria species, including *Borrelia*.

Important new zoonotic viruses are emerging and well known viral infections are resurging. Reviews were recently published on novel and emerging tick-borne viruses [86,87,118,119]. Tick-borne viruses are of increasing global medical and veterinary importance due to emerging and resurging viruses, expanding geographic ranges of tick vectors, and well recognized viruses appearing in new areas [86,87]. Severe fever with thrombocytopenia syndrome is an emerging hemorrhagic fever viral zoonosis caused by a phlebovirus of the family, Bunyaviridae [120]. Severe fever with thrombocytopenia virus was first described from patients in rural China [120] and the closely related Heartland virus was isolated from severely ill patients in northwestern Missouri in the United States [121]. Vector competent ticks for this important emerging virus are *Haemaphysalis longicornis* and *Rhipicephalus* (*Boophilus*) *microplus* in China [120] and *Amblyomma americanum* in the United States [46,122]. Geographic distributions of these ticks indicate that the severe fever with thrombocytopenia syndrome virus could be widely distributed. Heartland virus neutralizing antibodies were detected in vertebrate wildlife species from Texas to Maine, coinciding with the distribution of *Amblyomma americanum* [47]. Bourbon virus is a novel thogotovirus recently discovered in the Midwestern United States that is transmitted by *Amblyomma americanum* [48,123].

Well established tick-borne viruses are expanding their geographic areas of occurrence and/or resurging in incidence. Tick-borne encephalitis serogroup viruses are increasing their range across Europe [124], while Powassan virus is a re-emerging public health threat in North America [125,126]. Crimean-Congo hemorrhagic fever virus is occurring in new sites around the Mediterranean, including Spain, France, Italy, and Turkey, in addition to Africa, Asia, and eastern Europe where it is well established [127,128]. The extension of Crimean-Congo hemorrhagic fever into new areas is possibly based upon birds transporting the tick vector, *Hyalomma marginatum*, into Central Europe [129]. Alkhurma virus is a tick-borne hemorrhagic fever zoonosis linked to livestock in Saudi Arabia, with a potentially wider distribution than previously known [130]. Alkhurma hemorrhagic fever virus is a variant of Kyasanur virus, first detected in the 1950s in India and now thought to be more widely disseminated than the initial focus in the Karnataka state of India [87,131].

## 5. Reversed Discovery of Tick-Borne Diseases

The use of powerful molecular methods, such as high throughput sequencing, is increasing the identification in ticks of microorganisms that are not currently linked to human disease [65,118]. Tick microbiome characterizations provide detailed analyses of symbionts, known human and veterinary pathogens, potential causes of future zoonotic disease, and yet to be identified microbes associated with a tick [70].

The application of molecular techniques was the basis for the reversed discovery of tick associated microbes that were, subsequently, recognized as human pathogens: *Borrelia miyamotoi* [72], *Neoehrlichia mikurensis* [73], *Rickettsia helvetica* [132], *Rickettsia monacensis* [74], and other *Rickettsia* species identified by genomic methods [71]. High throughput sequencing of the microbiomes of *Ixodes scapularis*, *Dermacentor variabilis*, and *Amblyomma americanum* from a single site in New York State resulted in identification of nine new viruses [119]. This study design was expanded to multiple sites in Connecticut, New York, and Virginia, with the detection of nine previously characterized viruses and 24 presumably novel viral species [118]. New microbial species were detected in Western Europe when the *Ixodes ricinus* microbiome was analyzed by next generation sequencing [133]. Microbe DNA detected in a fed tick may be residual from a previous blood meal and not definitive evidence that the tick species in question is a competent vector for that microbe [134].

Expansion of tick species into new geographic areas presents the threat of the introduction of well characterized, resurging, and emerging tick-borne infectious agents into those regions. The characterization of microbiomes of tick species infesting humans in an area can provide a database of known and possible disease threats to public health officials, healthcare providers, and the public. Such tick associated microbe databases could prove invaluable in the identification of emerging tick-borne diseases.

## 6. Tick Saliva: Key to Blood Feeding Success and Pathogen Transmission

Tick saliva creates a host cutaneous environment favorable, and, potentially, essential, for successful blood feeding, as well as the transmission and establishment of tick-borne infectious agents by the suppression or deviation of host pain/itch responses, hemostasis, inflammation, innate and adaptive immune defenses, and wound healing [12,13,14,16,86,135,136,137,138,139,140,141,142,143]. The amazing complexity of tick salivary gland secretions was first appreciated by the analysis of cDNA library expressed sequence tags of multiple ixodid species of medical importance [15,144,145,146]. Significant differences in the encoded molecules of expressed salivary gland transcriptomes were found upon comparison of over 500 expressed proteins of *Ixodes scapularis* [144] and approximately 700 expressed proteins of *Dermacentor andersoni* [145]. Initial salivary gland transcriptomes characterizations were under estimates of protein constituent complexities with the application of high throughput sequencing technology [13,15,86]. Combined transcriptome and proteome analyses of salivary glands enhances the scope and depth of understanding of functional activities [15,16,147]. These studies reveal salivary glands are comprised of multiple gene families encoding molecules, with diverse biochemical activities, gene duplication and functional redundancies, and differential expression of genes during the course of blood feeding. Salivary gland transcriptomes also differ among individual ticks [148]. Non-protein saliva molecules are also modulators of host defenses [149].

The analysis of tick salivary glands revealed differential gene expression during infection with tick-borne pathogens; however, the roles, if any, of these differentially expressed genes in pathogen transmission and establishment within the skin at the tick bite site remains to be determined. Differential salivary gland gene expression was characterized for *Ixodes scapularis* nymphs infected with *Borrelia burgdorferi* [144]. Differential gene expression occurred in both the salivary glands and midgut of *Rhipicephalus microplus* when infected with *Anaplasma marginale* [150]. *Bartonella henselae* infection of *Ixodes ricinus* induced significant numbers of both upregulated and downregulated salivary gland genes, including within members of the same multigene family [151]. Interconnections of the salivary gland and other tick tissue transcriptomes were reviewed in the context of tick physiology, tick-host interactions, and pathogen transmission [15].

Recent reviews examined the activities of the saliva and individual saliva molecules of diverse species of ticks on individual cellular and molecular elements and pathways of vertebrate host inflammation, innate, and adaptive immune defenses [12,13,14,16,86,138,141,142,143]. The following overview of these ixodid tick-host relationships is presented as a model that examines defense modulating activities collectively across tick species on the normal host responses that would be expected to occur in response to injury created by a tick bite and how the tick modulated host environment creates cutaneous sites favorable for the success of tick transmitted infectious agents. Readers seeking up-to-date background material on inflammation and cellular and molecular immunology should consult the superb textbook by Abbas et al. [152].

Tick mouthparts are composed of two chelicerae, cutting plates that penetrate the epidermis into the dermis, and the ventrally positioned hypostome, which anchors the mouthparts into the skin and, in combination with the overlying chelicerae, forms a channel for the introduction of saliva and the uptake of blood from the pool-like feeding site [153,154]. Many ixodid species produce a protein attachment cement that is deposited in different configurations, depending upon the tick species, to help anchor the tick to the host skin [154]. Saliva is introduced during the attachment process directly into the bite site, percutaneously around the mouthparts, and into the attachment cement, exposing Langerhans cells and keratinocytes of the epidermis and the myriad of diverse cells in the dermis to the biological actions of the saliva molecules [155,156].

Tick saliva suppresses proinflammatory cytokine production by inhibiting the Toll-like receptor stimulation of keratinocytes, the most abundant basal cells in the epidermis [157]. Tick feeding reduces the number of Langerhans cells, epidermal dendritic cells, in the areas surrounding the bite during an initial infestation [158]. Tick saliva also inhibits keratinocyte production of the chemokines IL-8, a monocyte attractant protein, and antimicrobial peptide defensins [159]. At the epidermal level, these changes can reduce the early aspects of inflammation and antimicrobial responses, as well as antigen uptake and the processing and presentation by Langerhans dendritic cells that normally traffic to draining lymph nodes to stimulate a primary immune response. Suppressing keratinocyte proinflammatory cytokines can reduce dermal endothelial activation, attraction of neutrophils and macrophages to the tick bite site, and dendritic cell activation.

In general, innate immune effectors within the dermis are down regulated to reduce the development and expression of immunity to the foreign body, tick mouthparts, and saliva molecules introduced into the skin. Innate immune antivirus responses include type I alpha and beta interferons produced by a variety of cells and natural killer (NK) cells that directly kill virus infected target cells and produce macrophage activating interferon gamma, IFN-gamma [160,161]. Tick saliva inhibits interferon-beta production [162], as well as NK cell binding to and killing of target cells [163,164]. Inhibition of type I interferon can disrupt immune regulation and virus inhibiting responses in many cell types. Impaired NK cell function reduces innate and first lines of defense against introduced viruses.

Approximately ten years ago, innate lymphoid cells were recognized and their diverse roles in inflammation, autoimmunity, immune regulation, and other physiological processes began to be characterized [165,166,167,168,169]. Innate lymphoid cells are classified based on their similarities to cytotoxic, Th1, Th2, and Th17 T lymphocytes of the adaptive immune response; they lack somatically rearranged antigen receptors; their distribution includes nonlymphoid and lymphoid tissues; and, their activities include integration of innate and adaptive immunity [165,168,169]. The innate lymphoid cells subgroups, functionally, are homologous to adaptive immune response T lymphocyte populations in function and cytokine elaboration with cytotoxic innate lymphoid cells, including NK cells; group one innate lymphoid cells resemble Th1 lymphocytes; group two innate lymphoid cells share functional characteristics with Th2 lymphocytes; and innate lymphoid cells group three cells are compared to Th17 CD4+ T lymphocytes [165,169]. Innate lymphoid cells also express class II major histocompatibility antigen that suggests roles in immune regulation for these cells activated by a variety of cellular factors, including cytokines and other regulators of immune function [166,169]. Other than very limited examinations of NK cells, the roles of innate lymphoid cells in tick feeding and responses to tick-borne pathogens remain unexplored. Innate lymphoid cells can be assumed to be important contributors to immune responses at the tick-host-pathogen interface. Studying these relationships will likely be highly productive in providing new insights into these vectors and pathogens as they interact with their hosts.

The alternative pathway of complement is an integral component of innate immune defenses that is a rapidly activated series of proteins directed against invading microorganisms and damaged cells to promote their destruction, phagocytosis, and produce bioactive molecules with vasoactive and chemotactic properties [170,171]. Tick saliva blocks the alternative complement pathway, C3 convertase [172], by inhibiting factor B cleavage, as well as C3a anaphylatoxin formation and deposition of the important opsonizing factor, C3b [173]. Tick inhibition of the alternative complement pathway reduces a major innate immune effector that targets microbes for direct killing, inhibits microbe opsonization, and reduces chemoattraction of inflammatory cells. Alternative complement pathway suppression reduces the amplification pathway activated in response to classical complement pathway activation and reduces host defenses against the tick and tick-borne infectious agents.

Macrophages are described as resident or circulating cells within tissues and as proinflammatory, classically activated M1, or anti-inflammatory, alternatively activated M2 populations [174,175]. Tick saliva suppresses macrophage proinflammatory cytokines’ production [176,177], antimicrobial nitric oxide [178], and the Th1 polarizing cytokine IL-12 and immune synapse costimulatory molecules [179]. These changes in macrophage function can reduce hematopoiesis of granulocytes, polarization of the helper T lymphocyte response, and signaling at the immune synapse of the antigen presenting cell and T lymphocyte.

Suppression of proinflammatory cytokines and chemokines is a common feature of the saliva of many tick species [12,138]. Proinflammatory cytokines activate post-capillary endothelial cells to express selectins and other adhesion molecules, and, in concert with chemokine interactions with leukocyte integrins, orchestrate leukocyte egress from the vascular compartment into tissues at sites of injury and/or infection [180,181]. Tick saliva down regulates the expression of E-selectin, P-selectin, ICAM-1, VCAM-1, and the CD18 component of leukocyte surface, β2-integrin [182,183,184]. Collective down regulation of endothelium activation, adhesion molecule expression, chemokines, and, thus, integrin transition to a high affinity state by chemokines creates an environment where accumulation of inflammatory cells is suppressed and a site more favorable to successful tick feeding and pathogen establishment is created. Histologic examination of tick bite sites reveals a very minimal inflammatory cell response during a first infestation and a greatly increased inflammatory response throughout the bite site and surrounding the tick mouthparts during a repeated infestation [185].

Neutrophils are the first cells recruited to an inflammatory focus where they phagocytose and kill extracellular pathogens, activate and recruit other cells, and both cause tissue damage and participate in tissue repair [186,187]. Tick saliva diintegrin metalloproteinases reduces β2-integrin expression involved in neutrophil adherence for movement into tissues at the bite site, superoxide production, and killing of *Borrelia burgdorferi* [188].

Dendritic cells are heterogeneous populations of cells that are central regulators of adaptive immunity by orchestrating naïve T cell immune responses and by acting as an interface between innate and adaptive immunity [189]. With such pivotal roles in immune regulation, it is not surprising that ticks modulate dendritic cell functions. Although an initial tick infestation reduced Langerhans cell numbers around tick mouthparts, repeated infestation increased their numbers at tick bite sites for hosts expressing an acquired resistance to infestation [158]. Tick saliva reduces bone marrow derived dendritic cell development, differentiation, and maturation; diminishes costimulatory molecule expression at the immune synapse of dendritic cells and T lymphocytes; impairs chemokine receptor expression and chemotactic responses; and inhibits the Th1 polarizing cytokine, IL-12, as well as proinflammatory cytokine production, while enhancing the expression of the anti-inflammatory cytokine, IL-10 [190,191,192,193,194].

Tick saliva modifies dendritic cell responses to tick-borne pathogens. *Ixodes ricinus* saliva reduced dendritic cell uptake of *Borrelia afzelli* that would reduce subsequent antigen presentation [195]. Dendritic cell apoptosis is reduced and frequency of cell infection increased with tick-borne encephalitis virus upon exposure to *Ixodes ricinus* saliva [196]. *Ixodes scapularis* saliva, sialostatin L2, reduces Toll-like receptor induced dendritic cell signal transduction pathway activation during *Borrelia burgdorferi* infection [197]. Additional studies of the interactions among tick saliva, tick-borne pathogens, and dendritic cells are needed, including the impact of repeated infestations on dendritic cell functions.

T lymphocyte subsets are the predominate cellular effectors and regulators of cutaneous adaptive immune responses [198,199]. There are approximately twice as many circulating T lymphocytes as resident T cells in the skin, and the circulating T cells are predominantly of an effector Th1 phenotype [200]. Cutaneous T lymphocyte populations include resident populations of memory regulatory T cells and effector memory cells, as well as cytotoxic, CD8+ T lymphocytes that receive CD4+ helper T cell growth signals, such as IL-2 [201,202].

The fact that ticks target a myriad of T lymphocyte effectors and regulatory activities is not surprising. Tick inhibition of T lymphocyte proliferation [12,13,203] is a common host defense modulating strategy linked to multiple saliva proteins in diverse tick species [204,205]. T lymphocyte proliferative responses are further inhibited by tick saliva IL-2 binding protein and inhibition of T lymphocyte IL-2 production [205,206]. Another common tick saliva induced host immunomodulation strategy is polarization of CD4+ T helper lymphocytes to a Th2 response, accompanied by the production of IL-4, IL-5, IL-6, and IL-10, with concomitant suppression of Th1 responses and cytokines, including interferon-γ [12,13,138,141,143]. Suppression of interferon-γ reduces the ability to activate macrophages, an important aspect of killing intracellular pathogens, providing help to B lymphocytes for class switching to IgG antibody production, and for the formation of granulomas. Tick saliva also inhibits cytotoxic T lymphocyte proliferation [207]. Suppressed helper T lymphocytes and reduced IL-2 availability will impair cytotoxic T lymphocytes, since helper T lymphocytes are the primary source of IL-2 that is essential for cytotoxic T lymphocyte proliferation.

Tick salivary gland molecules suppress B lymphocyte proliferation and, in one case, were linked to a specific saliva protein [126,208,209]. Tick infestation reduced both a primary IgM and IgG antibody responses to heterologous antigens [210,211,212]. The down regulation of antibody responses could result from the direct effects of tick saliva on B lymphocytes and/or the suppression of helper T lymphocytes and their mediators that are essential for antibody class switching to IgG and other isotypes.

Although individual tick species were not linked to each of the host immunomodulatory phenomena just described, the scope of how ticks down regulate and deviate host inflammation, innate, and adaptive immunity are impressive. Tick countermeasures to host pain and itch, hemostasis, and immune defenses likely evolved so that ticks of a specific species could repeatedly obtain blood meals during the life of the host; however, for some tick-host associations, acquired resistance to tick feeding develops [12,13,136,137,143,203]. Tick modification of the host cutaneous interface provides a favorable, and possibly privileged, site for the introduction and establishment of tick-borne infectious agents. This author believes that the repertoire of pathogens transmitted by a specific tick species is dependent, in large, upon the host immunosuppressive properties of the saliva of the vector tick [12,137].

## 7. Concluding Thoughts

Public awareness of the increasing population sizes, expanding geographic ranges of important tick vectors, and emergence and resurgence of tick-borne infectious agents is becoming greater. There exists the potential to develop integrated surveillance strategies for monitoring tick species’ movements into new regions and changes in their population sizes that is combined with high throughput sequencing of tick microbiomes. Central to these efforts will be further microbiome gene annotations to define both tick symbionts and, potentially, transmitted microbes. Microbiome databases for ticks of medical and veterinary importance will provide physicians, veterinarians, public health works, other stakeholders, and the public with current information about recognized and, potentially, emerging tick-borne infectious agents that threaten, or could threaten, a region. Information obtained from these ongoing analyses will help with planning and assessing control interventions and protecting the health of companion, domestic, and wildlife animal species, as well as humans. Changes in tick geographic distributions and numbers, and the emergence of new tick-borne infectious agents, will continue. We need to be knowledgeable of those changes. A One Health approach is highly appropriate, if not essential, to be prepared to protect against these zoonotic infectious agents.

Significant advances are being made, at an increasingly rapid pace, in understanding the tick-host-pathogen interface. Studies with multiple tick species and tick-borne pathogens established the importance of tick modulation of host pain/itch, hemostasis, inflammatory responses, innate and adaptive immunity, and wound healing. The complexity of saliva protein and non-protein components are increasing, with greater awareness of families of bioactive molecules, redundancies in activities, and differential expression during tick feeding. Linking specific saliva molecules to specific defense modulatory activities remains a major challenge; however, progress is being made toward achieving that goal and dissecting the underlying cellular and molecular mechanisms involved.
